# Defensive Medicine in Israel – A Nationwide Survey

**DOI:** 10.1371/journal.pone.0042613

**Published:** 2012-08-16

**Authors:** Elad Asher, Sari Greenberg-Dotan, Jonathan Halevy, Shimon Glick, Haim Reuveni

**Affiliations:** 1 Sheba Medical Center, Tel Hashomer, Ramat-Gan, Israel; 2 Ben-Gurion University of the Negev, Beer-Sheva, Israel; 3 Shaare Zedek Medical Center, Jerusalem, Israel; Yale University School of Medicine, United States of America

## Abstract

**Background:**

Defensive medicine is the practice of diagnostic or therapeutic measures conducted primarily as a safeguard against possible malpractice liability. We studied the extent, reasons, and characteristics of defensive medicine in the Israeli health care system.

**Methods and Findings:**

Cross-sectional study performed in the Israeli health care system between April and July 2008 in a sample (7%) of board certified physicians from eight medical disciplines (internal medicine, pediatrics, general surgery, family medicine, obstetrics and gynecology, orthopedic surgery, cardiology, and neurosurgery). A total of 889 physicians (7% of all Israeli board certified specialists) completed the survey. The majority [60%, (95%CI 0·57–0·63)] reported practicing defensive medicine; 40% (95%CI 0·37–0·43) consider every patient as a potential threat for a medical lawsuit; 25% (95%CI 0·22–0·28) have previously been sued at least once during their career. Independent predictors for practicing defensive medicine were surgical specialty [OR = 1.6 (95%CI 1·2–2·2), *p* = 0·0004], not performing a fellowship abroad [OR = 1·5 (95%CI 1·1–2), *p* = 0·027], and previous exposure to lawsuits [OR = 2·4 (95%CI 1·7–3·4), *p*<0·0001]. Independent predictors for the risk of being sued during a physician's career were male gender [OR = 1·6 (95%CI 1·1–2·2), *p* = 0·012] and surgery specialty [OR = 3·2 (95%CI 2·4–4·3), *p*<0·0001] (general surgery, obstetrics and gynecology, orthopedic surgery, and neurosurgery).

**Conclusions:**

Defensive medicine is very prevalent in daily physician practice in all medical disciplines. It exposes patients to complications due to unnecessary tests and procedures, affects quality of care and costs, and undermines doctor-patient relationships. Further studies are needed to understand how to minimize defensive medicine resulting from an increased malpractice liability market.

## Introduction

Defensive medicine is defined as the ordering of tests, procedures, and visits, or the avoidance of high-risk patients or procedures, primarily to reduce exposure to malpractice liability [1]. Many physicians practice defensive medicine, and this phenomenon is believed to influence all medical fields. Moreover, to avoid lawsuits non-evidence-based procedures are frequently used. This has become deeply ingrained in many physicians' practices resulting in “unconscious" defensive medicine [2]. Defensive medicine is expensive, has no basis in evidence-based studies, and sometimes exposes patients to complications due to unnecessary tests and procedures [3]. The cost of defensive medicine in the United States is estimated to be as high as $50 billion annually, more than the costs for the treatment of hypertension and chronic obstructive pulmonary disease combined [2], [3]. Defensive medicine has been reported in the United States and elsewhere [1], [2], [4]. Its prevalence was found to be as high as 93% among high-risk specialty physicians in Pennsylvania [5]. Moreover, 6 specialties (emergency medicine, general surgery, neurosurgery, obstetrics/gynecology, orthopedic surgery, and radiology) were identified as being especially affected by high and rising liability costs [5]. In Israel data about defensive medicine is lacking. Only small studies issued this phenomenon and none of them was related to the extent of it [2], [6], [7]. Defensive medicine prevalence and characteristics remain controversial [8], [9] and to date no nationwide survey has been done to evaluate this phenomenon. In this study we performed for the first time a nationwide study to measure the extent and characteristics of defensive medicine among Israeli board certified expert physicians from high- and low-risk specialties.

## Methods

### Israeli Healthcare and Malpractice Systems

Israel has maintained a system of socialized health care since its establishment in 1948. In 2010, there were 25,542 doctors in Israel – 3·36 doctors for every 1,000 people. Since 1995 all Israeli citizens are entitled by law to the same health care Uniform Benefits Package, for example: medical diagnosis and treatment, preventive medicine, hospitalization (general, maternity, psychiatric, and chronic), surgery and transplants, preventive dental care for children, and first aid. In cross-country comparisons between health care systems in the Organization for Economic Co-operation and Development (OECD) countries, the Israeli healthcare system was similar to the quality of care and technologically advanced to these countries. The Israeli malpractice system is somewhat similar to the U.S. malpractice system and there are no features like a no-fault system. In the previous few years, there were between 4200–4500 medical lawsuits in Israel every year and the average compensation for every patient was $62,000 in the years 2008/9 compared to only $22,000 5 years earlier [10]. Researchers from the Faculty of Health Sciences and the Guilford Glazer School of Business and Management at the Ben-Gurion University of the Negev collaborated with the Israel Medical Association (IMA) to conduct the study. The IMA is the official organization representing the physicians in Israel. The survey was supported by the Israel national institute for health policy research.

### Study Design and Population

A stratified random sample of board-certified expert physicians (aged 28–65 years) from eight major medical disciplines: internal medicine, pediatrics, general surgery, family medicine, obstetrics and gynecology, orthopedic surgery, cardiology, and neurosurgery, was drawn from the IMA data bases. A computer program randomly sampled every 7th physician in specialties with more than 1000 physicians, every 5th physician in specialties with 500–1000 physicians, and every 2nd physician in specialties with fewer than 500 physicians. At least 8% of the physicians in each specialty were sampled with the exception of neurosurgeons, who were over sampled [31 of 66 (47%)] to ensure adequate representation due to the small number of neurosurgeons in Israel. The sample size was calculated to provide 80% power to detect differences of 10% or more between specialty groups at the *p*<0·05 level. Three surveyors conducted the calls from the IMA office after having participated in a teaching and practice session. Responders and non-responders were statistically similar with regard to gender distribution and average age. According to the academic medical center at the Ben Gurion University, ethical approval for this study was deemed unnecessary since this study was a physician's survey and no patient was included. Every physician was verbally consented to participate in the survey at the time of the telephone interview.

### Survey Development and Administration

A seven-page, telephone-administered, confidential questionnaire was developed by a team of board certified physicians, lawyers, and specialists in health systems management and ethics. Some questions were translated from a previous questionnaire, after permission of the corresponding author [5]. For validation, a preliminary study was conducted among 20 specialist physicians. After comments, the final questionnaire was approved. None of the 20 physicians used in the validation participated in the study. The final questionnaire contained questions addressing demographics, occupation, insurance and liability, experience with malpractice claims, direct and indirect questions about defensive medicine, reasons for performing defensive medicine, and questions regarding ways to minimize defensive medicine. Physicians were asked to rate their answers on a 5-point scale (1 – lowest, 5 – highest). The survey is proprietary and therefore cannot be made publicly available.

### Survey Content and Main Outcome Measures

All Physicians were asked about their concerns regarding malpractice liability and whether it caused them to act in each of four forms of “assurance" behavior: (1) order more tests than medically indicated; (2) prescribe more medications than medically indicated; (3) refer to specialists in unnecessary circumstances; and (4) suggest invasive procedures against professional judgment. Physicians used the same 5 point scale to rate the frequency with which they practiced two forms of avoidance behavior: (1) avoid conducting certain procedures/interventions and (2) avoid caring for high-risk patients. Physicians who reported engaging in any of these defensive medicine practices were then asked in an open-ended question to describe their most recent act of defensive medicine. Finally, physicians were asked about ways to minimize the need to practice defensive medicine.

### Statistical Analysis

Two logistic regression models, based on enter methods, were conducted. Outcomes of each were:

1. “Sued previously for medical malpractice" – this variable includes all physicians who had previously been sued.
2. “Practices defensive medicine" – was based on a direct question “In order to avoid complaints and lawsuits, do you practice defensive medicine?"

The independent variables were included due to significant results in a univariant analysis that was established as a first stage, or due to their potentially impact on the relationship between the outcome variable and the main independent variables. Predictor's variables were: age, gender, professional satisfaction, place of medical school graduation, private malpractice insurance, previous exposure to a lawsuit, main practice location, performing a fellowship outside of Israel and type of specialty. All tests were two-sided, and values of *p*<0·05 were considered statistically significant, using SPSS 15 (SPSS Inc., Chicago, IL).

## Results

### Physician Characteristics

During a four-month period, a total of 1800 physicians were approached, of whom 889 completed the survey (50% response rate). Table 1 presents physician characteristics. Twelve physicians who practice only medical administration were excluded. The remaining 877 (98.7%) comprised the study population, representing 7% of all Israeli board certified specialists. Of them, 640 (73%) were men; mean age was 51·29 years (median 53 years). Five hundred sixty-seven physicians (65%) graduated in Israel, 94 (11%) in the former Soviet Union, 64 (7%) in Italy and 152 (17%) in other countries. Responders and non-responders were similar (*p* = 0·5) in terms of age and sex. Of the 877 physicians included in the final analysis, 189 (22%) were pediatricians, 169 (19%) were internists, 127 (14%) were obstetricians and gynecologists, 112 (13%) were orthopedic surgeons, 111 (13%) were family medicine practitioners, 82 (9%) were general surgeons, 56 (6%) were cardiologists, and 31 (4%) were neurosurgeons. Three hundred and forty-seven (40%) physicians completed a fellowship outside Israel. The main practice locations were hospital 428 (49%) ambulatory clinics 268 (31%) a combination of hospital and ambulatory clinics 169 (19%) and other places 12 (1%).

**Table 1:** Physicians' Characteristics.

Characteristic	No. (%) of Physicians (N = 877)
**Specialty**	
Pediatrics	189 (22%)
Internal medicine	169 (19%)
Obstetrics and gynecology	127 (14%)
Orthopedic surgery	112 13%)
Family medicine	111 (13%)
General surgery	82 (9%)
Cardiology	56 (6%)
Neurosurgery	31 (4%)
**Sex**	
Male	640 (73%)
Female	237 (27%)
**Received degree**	
Israel	579 (66%)
Former Soviet Union	94 (11%)
Italy	64 (7%)
Hungary	6 (<1%)
United States	5 (<1%)
Other	141 (16%)
**Fellowship**	
Outside of Israel	347 (40%)
Did not have fellowship abroad	530 (60%)
**Main Place of Practice**	
Hospital	428 (49%)
Ambulatory clinics	268 (31%)
Hospital and ambulatory clinics	169 (19%)
Other	12 (1%)
**Medical Insurance**	
Malpractice insurance provided by employer	757 (86%)
Private malpractice insurance	8 (1%)
Private malpractice insurance in addition to employer-provided insurance	109 (12%)

#### Working as physicians

The majority of the physicians 808 (92%) was satisfied or very satisfied with their profession. Overall, 431 (49%) claimed it was hard to find new fellowship candidates in their field of expertise. Only 188 (21%) thought that the reason had to do with defensive medicine (of them, 40% are gynecologists). One hundred and seventeen physicians (13%) stated that at least one candidate for fellowship had told them he was concerned about the liability atmosphere in medicine (of them, 45% gynecologists).

### Malpractice insurance policy

When working in the public sector (hospitals and health maintenance organizations-HMO's), physicians had malpractice insurance as part of employment contracts, which includes private practice. Seven hundred and sixty (87%) had their malpractice insurance provided by their employer, 109 (12%) had private malpractice insurance in addition to their employer insurance, and 8 (1%) had only a private malpractice insurance policy. The majority 603 (69%) believed that the malpractice insurance would cover all of their needs in case of a malpractice lawsuit.

#### Exposure to medical lawsuits

**Figure 1:**
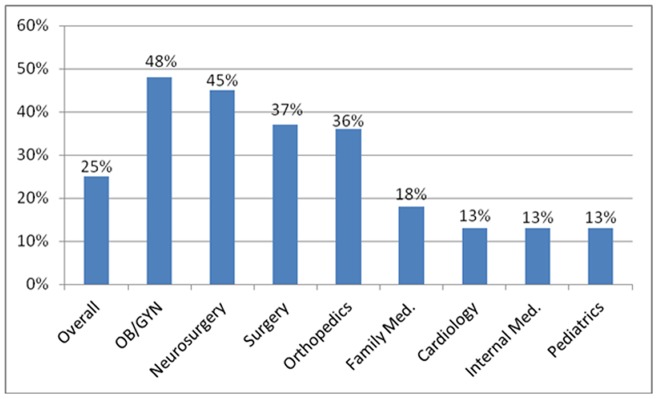
Percentage of board certified physicians (n = 219) exposed to lawsuits in each of the eight studied specialties.

**Figure 2:**
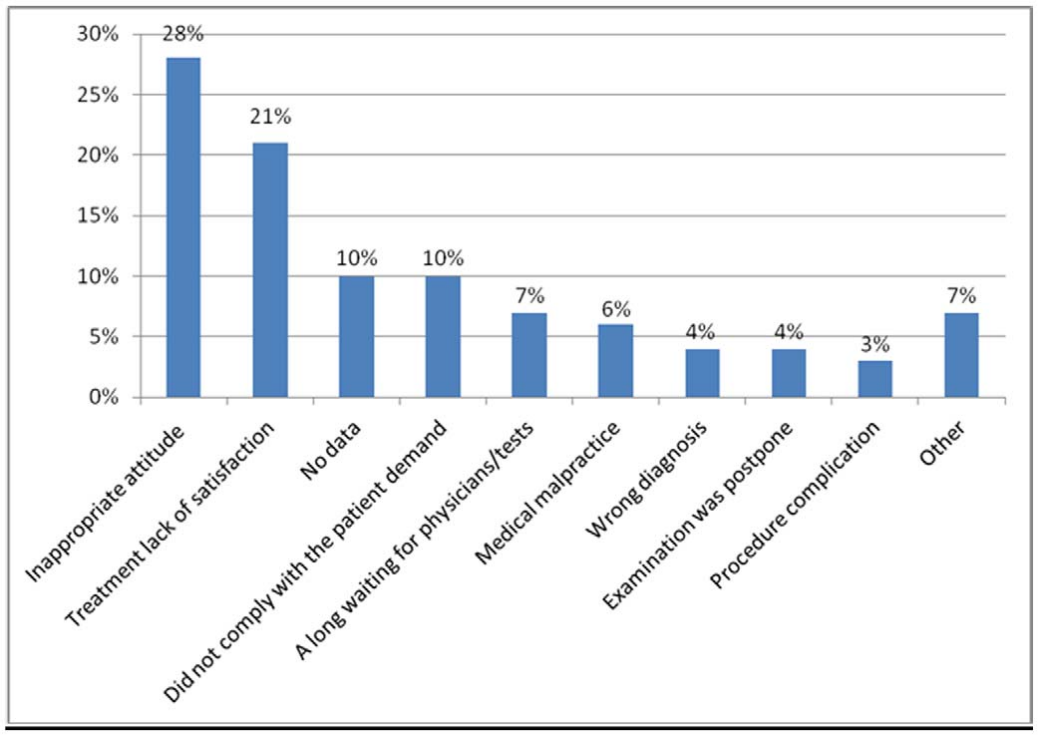
Main reasons for complaint letters against physicians (n = 233).

Two hundred and nineteen [25% (95% CI 22%, 28%)] participating physicians have previously been sued at least once during their career. In addition, the names of 253 physicians [29% (95% CI 26%, 32%)] have been mentioned in indirect lawsuits in the past, and complaint letters have been served against 233 [27% (95% CI 24%, 30%)] in recent years. Figure 1 shows the percentage of board certified physicians (n = 219) exposed to lawsuits in each of the eight studied specialties. Figure 2 describes the main reasons for complaint letters.

### Defensive medicine and daily practice

The majority of the physicians [756, 86%, (95% CI 84%, 88%)] claimed that the increased rate of medical lawsuits in Israel prevents them from giving their patients the best medical treatment possible. Moreover, 64 [7%, (95% CI 6%, 9%)] reported stopping their practice or eliminating high-risk procedures three years before the survey due to fear of medical lawsuits. One hundred and fifty-seven physicians [18% (95% CI 16%, 20%)] think there is a possibility that in the next few years they would stop practicing or eliminate high-risk procedures due to fear of medical lawsuits and 69 [8% (95%CI 6%, 10%)]consider stopping direct patient care entirely for the same reason. Table 2 describes the assurance and avoidance behaviors of physicians practicing defensive medicine in daily practice.

**Table 2:** Defensive medicine in daily practice (n = 877 physicians).

Practice defensive medicine to greater extent in recent years	60% (95%CI 0.57–0.63)
Perform more tests (e.g., blood tests; chest x-rays; brain CT) due to defensive medicine	59% (95%CI 0.56–0.62)
Refer more patients to consultants in unnecessary circumstances	50% (95%CI 0.47–0.53)
Admit more patients unnecessarily	30% (95%CI 0.27–0.33)
Suggest invasive procedures (e.g., biopsies) to confirm diagnoses	24% (95%CI 0.21–0.27)
Prescribe more medications than indicated	12% (95%CI 0.1–0.14)
Avoid certain procedures or interventions	17% (95%CI 0.15–0.2)
Stop practicing or eliminate high-risk procedures	7%.(95%CI 0.05–0.09)

CT – computerized tomography.

Five hundred and twenty-six [60% (95% CI 57%, 63%)] physicians admitted to practicing defensive medicine. Almost half of the physicians [347, 40% (95% CI 36%, 43%)] consider every patient as a potential threat for a medical lawsuit. One hundred eighty-two [21% (95% CI 18%, 24%)] are less frank with their patients due to defensive medicine, and almost all study participants [813, 93% (95% CI 90%, 94%)] think patients and their families have become more demanding in recent years. Seven hundred twelve physicians [81%, (95% CI 78%, 84%)] think that the recently developed culture of high lawsuit and compensation rates influence the Health Maintenance Organizations' (HMOs) policy. Financially, 484 [55% (95% CI 52%, 58%)] described using more health care resources, resulting in higher medical costs due to defensive medicine. Seven hundred and thirty-four [84% (95% CI 81%, 86%)] stated that this cost will not prevent them from practicing defensive medicine in the future (see Tables 3 and 4).

**Table 3:** Determinants of “Sued previously for medical malpractice" in a multivariate analysis.*

Variable	OR (95% CI)	Pv
Surgical specialty	3 (2.2–4.2)	<0.0001
Gender (men)	1.6 (1.1–2.2)	0.012
Age (≥50 years)	1.4 (0.97–2)	0.077
Fellowship outside of Israel	0.93 (0.66–1.3)	0.661
Graduated in Israel	0.96 (0.7–1.3)	0.822
Work in hospitals	0.96 (0.68–1.4)	0.825
Managerial job	1.2 (0.9–1.7)	0.201
Professional satisfaction	1.01 (0.61–1.8)	0.870
Private insurance	0.96 (0.63–1.5)	0.848

* Logistic regression.

**Table 4:** Determinants of “Practices defensive medicine" in a multi variant analysis.*

Variable	OR (95% CI)	Pv
Surgical specialty	1.6 (1.2–2.2)	0.004
Fellowship outside of Israel	1.5 (1.1–2)	0.027
Previous exposure to a lawsuit or to complaint letters	2.4 (1.7–3.4)	<0.0001
Age (≥50 years)	0.73 (0.53–0.99)	0.048
Gender (men)	0.94 (0.66–1.3)	0.717
Graduated in Israel	0.84 (0.62–1.2)	0.277
Working in hospital	0.72 (0.51–1.01)	0.060
Managerial job	1.05 (0.76–1.5)	0.749
Professional satisfaction	0.79 (0.46–1.34)	0.376
Private insurance	0.91 (0.59–1.4)	0.647

* Logistic regression.

### Ways to reduce lawsuits and complaints

**Figure 3:**
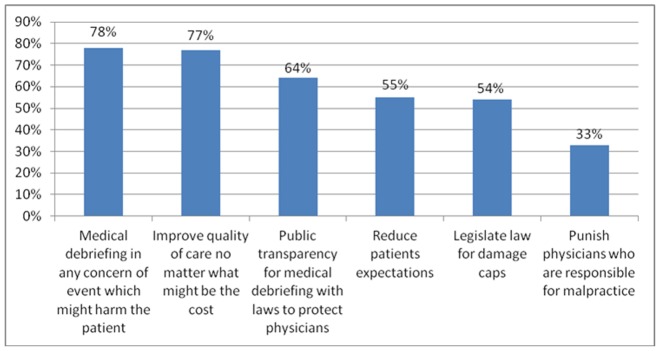
Physicians' suggestions to reduce lawsuits and complaints.*

When asked about ways to reduce lawsuits and complaints, and hence defensive medicine, most study participants suggested medical debriefing in any event that might harm the patient, and improving the quality of care. Nevertheless, when asked if in today's medico-legal reality, they think they should report medical care mistakes to an internal working place committee, 456 physicians [52% (95% CI 49%, 55%)] replied they would do so only if they would be offered anonymity, or if they would be provided with lawsuit immunity, otherwise they would not be willing to report such mistakes in any case (See Figure 3).

### Multivariate logistic regression model analysis

Multivariate logistic regression model analysis showed that only surgery specialty [OR = 3 (95% CI 2·42–4·2), *p*<0·0001] (general surgery, obstetrics and gynecology, orthopedic surgery, and neurosurgery) and male gender [OR = 1·6 (95%CI 1·1–2·2), *p* = 0·012] were independent predictors for the risk of being sued during a physician's career. Independent predictors for practicing defensive medicine were surgical specialty [OR = 1·6 (95%CI 1·2–2·2), *p* = 0·0004], not performing a fellowship outside of Israel [OR = 1·5 (95%CI 1·1–2), *p* = 0·027], and previous exposure to a lawsuit [OR = 2·4 (95%CI 1·7–3·4), *p*<0·0001].

## Discussion

This is the first nationwide survey regarding the practice of defensive medicine performed among board certified expert physicians from eight medical disciplines (surgical and non-surgical). Previous surveys have focused on physicians subjected to relatively low levels of litigation [11]–[13] and were often limited to single specialties, such as obstetrics and gynecology [14], [15]. Another large survey was conducted in Pennsylvania among 824 high risk specialist physicians in a volatile malpractice environment [5].

In the current study, we included four major disciplines of medicine and four specialties that are at greater risk of litigation. Our study was not limited to a certain area or a single specialty, nor was it limited to high risk or low risk specialists alone.

### The extent of defensive medicine in Israel

Sixty percent of physicians were routinely practicing defensive medicine. These findings are similar to the reported prevalence of defensive medicine in Australia [16], England [4] and the United States [5], where as many as 46%, 75%, and 93% of physicians, respectively, reported practicing defensive medicine. In Australia and England the surveys were limited to only one specialty- general practitioners, whereas in the United States the survey included six specialties (emergency medicine, general surgery, orthopedic surgery, neurosurgery, obstetrics/gynecology, and radiology), but was limited to Pennsylvania and was conducted in a volatile malpractice environment. These results suggest that defensive medicine is a well-embedded medical practice in industrialized countries. At least 50% of the physicians from every western country surveyed practice defensive medicine [1], [2], [4]–[5], [12]–[13], [16]–[17].

### Impact of defensive medicine on patient treatment

Little is known about whether defensive medicine reduces or improves quality of care. In our study, physicians were ordering unnecessary diagnostic tests, referring patients to unnecessary consultants, and hospitalizing patients unnecessarily. Simple diagnostic testing, such as blood tests, may be annoying but are not harmful to most patients. However, invasive procedures such as catheterizations and procedures involving radiation, as computed tomography, can cause radiation exposure and serious adverse effects [18], [19].

Of greater concern is the fact that 40% of physicians consider every patient they treat as a potential threat for a medical lawsuit, and as a result, 21% stated they are less frank with their patients. Some perform unnecessary procedures such as caesarean sections in order to avoid lawsuits; some are forced to perform life resuscitations and intubations, by senior physicians in their department, on patients that had a poor prognosis in order to avoid litigation by patients' family members. Therefore, it is not surprising that 81% of physicians think the recent culture of frequent lawsuits and compensation rates influence HMOs' policies.

### Defensive medicine and physician behavior

One out of four physicians had previously been sued during their career; one out of three received a complaint letter within the two years prior to the survey. Not surprising is the fact that physicians' liability experience and exposure were the most prominent single independent predictor for practicing defensive medicine. These results suggest that some patients who are seen as demanding, emotional, or unpredictable may prompt physicians to behave defensively. We found support for our findings in the United States, where similar practice patterns have been reported in teaching behaviors because of concerns about malpractice litigation [20], [21]. As a result, physicians' past experience of liability affects clinical decision-making processes and influences individual physicians' propensity to practice defensively [5], [21]–[24]. Nevertheless, in the United States, physicians' liability experience was not a predictor for practicing defensive medicine [5]. This major difference might be explained by the fact that our study population represents the major disciplines in medicine and not only high risk specialist physicians working in a volatile malpractice environment who are more concerned about liability, and hence practice defensive medicine regardless of previous lawsuits. Moreover, the situation in Israel, which is presented in the paper, is that the malpractice fee is paid by the Institutions and not by the doctors out of pocket. Therefore, the defensive medicine that is practiced in Israel is not mainly for financial reason. Our findings support a recent study from the United States where it was estimated that by the age of 65 years, 75% of physicians in low risk specialties would have faced a malpractice claim, compared with 99% of physicians in high-risk specialties [25].

### Ways to reduce lawsuits and complaints

Creating solutions to reduce the practice of defensive medicine is a challenging task, mainly because reliable empirical evidence on the subject remains limited. Defensive behaviors may reduce access to care and even cause physical harm [26]–[30]. Most physicians in our study suggested that reduced numbers of lawsuits and complaints may occur in the presence of medical debriefing in every medical event that might harm the patient and by improving the quality of care no matter what the costs are. Nevertheless, when asked if in today's medico-legal reality they would report medical mistakes, the majority of physicians replied they would not do so, probably because they do not trust the system. The literature regarding solutions for this phenomenon is poor [27]. Moreover, no study, to the best of our knowledge, has examined ways to deal with defensive medicine. The nature of its causes and effects are the subject of constant debate. Though many think that malpractice reform will reduce defensive medicine, they often disagree, due to lack of evidence, about which methods of such reform would be most effective and appropriate [28].

### Study limitations

Objective methods for measuring defensive medicine are almost impossible [1], [2], [9] because distinctions between inappropriate and appropriate care are not clear in many clinical situations [1], [5], [29]. It is also difficult to identify the difference between liability-related motivators and other factors that influence clinical decision making. Another limitation is that physician self-reports of defensive medicine may be biased, and may lead physicians to overstate the frequency of performing defensive medicine. In contrast, unconscious defensive medicine is not reported by physicians but it is also widely practiced [1], [2]. Nevertheless, our nationwide survey of physicians from eight medical disciplines (surgical and non-surgical) represents the best possible picture concerning defensive medicine in a single country.

### Conclusions

Defensive medicine is very prevalent in Israel. It is more prevalent among surgical specialists and among physicians with previous exposure to lawsuits. The most frequent daily practice of defensive medicine is performing more unnecessary tests and referring more patients to consultants and hospitalization. This study should provoke researchers to study how to minimize defensive medicine in terms of cost and quality of care, and regain trust in physician-patient relationships.
